# Microbial Dynamics and Functional Shift During Spontaneous Fermentation of Bee Pollen from Different Geographical Origins

**DOI:** 10.3390/microorganisms14081638

**Published:** 2026-07-27

**Authors:** Silvia Gattucci, Laura Canonico, Alice Agarbati, Maurizio Ciani, Francesca Comitini

**Affiliations:** 1 Department of Life and Environmental Sciences (DiSVA), Polytechnic University of Marche, Via Brecce Bianche, 60131 Ancona, Italy; s.gattucci@pm.univpm.it (S.G.); l.canonico@univpm.it (L.C.); a.agarbati@univpm.it (A.A.); 2 Department of Agricultural, Forest and Food Sciences, University of Turin, Via Verdi 8, 10124 Turin, Italy

**Keywords:** bee pollen, bee bread, yeasts, lactic acid bacteria, functional consortium

## Abstract

Bee bread is a fermented product derived from bee pollen, whose fermentation improves preservation, nutrient bioavailability, and functional properties. However, the microbial succession driving this process, particularly the role of yeasts, remains poorly understood. This study investigated microbial dynamics during fourteen-day spontaneous fermentation of five fresh bee pollen samples from different geographical origins, mimicking natural bee bread formation. Cultivable yeasts and lactic acid bacteria were monitored by viable cell counts and molecular identification. Physicochemical parameters, nutritional components, bioactivities, and pollen structure were evaluated. A clear microbial succession was observed, with *Starmerella* sp. dominating the early stages and the osmotolerant yeast *Zygosaccharomyces rouxii* prevailing during the final phase. Together with *Apilactobacillus kunkeei*, these microorganisms may constitute a cultivable fermentative core involved in bee bread formation. Fermentation significantly increased protein availability, with increases of up to 18%, reduced pH, promoted bee pollen degradation up to 16%, and enhanced antimicrobial activity against *Staphylococcus aureus* and *Listeria monocytogenes*. Understanding of cultivable microbiota dynamics during spontaneous bee pollen fermentation could provide an effective natural strategy to stabilize bee pollen while improving its nutritional and functional properties, laying the foundation for developing controlled industrial fermentations to produce bee bread-like products with consistent quality and health-promoting characteristics.

## 1. Introduction

Honey represents the most widely consumed honeybee product, followed by bee pollen, propolis, royal jelly, and wax. Recently, another derivative has gained significant consumer interest due to its antioxidant, anti-inflammatory, antiviral, antitumoral, probiotic, and prebiotic properties: “bee bread” [1,2,3,4,5]. Bee bread is the result of a successful evolutionary strategy by honeybees, which, through fermentation, created a food with multiple beneficial features, including long-term storage. This stability is due to the microbial activity that determines low pH, inhibits spoilage, and enhances the bioavailability of nutrients through the breakdown of the pollen wall, serving as the main food source for the brood [6,7].

The transformation from bee pollen to bee bread occurs in one to two weeks inside the honeycombs, depending on temperature and inoculum concentration, performed by honeybees, which regurgitate the contents of their crop, mainly containing lactic acid bacteria (LAB), yeasts, and enzymes, onto the freshly collected bee pollen [1,8,9]. According to Gilliam [10], the fermentation process unfolds in four distinct stages. The first phase, lasting approximately 12 h, is characterized by the proliferation of a heterogeneous microbial community comprising yeasts, LAB, and indole-producing bacteria (such as *Escherichia coli*), serving as a precursory stage for fermentation. In the second step, anaerobic LAB belonging to the genus *Streptococcus* utilize yeast-derived metabolites as growth factors, thereby reducing the pH. Subsequently, a specific group of fructophilic lactic acid bacteria (FLAB) flourishes, further acidifying the pollen to a pH of approximately 3.5–4.0. This rising acidity ultimately curtails LAB growth due to these stringent conditions. These pathways highlight the coordinated microbial activity required to transform pollen into a stable, nutrient-rich substrate [1,10].

The predominant yeasts inhabiting bee bread belong to the genera *Starmerella*, *Zygosaccharomyces*, *Metschnikowia* and *Debaryomyces*, although their relative abundances vary depending on the freshness of the product. Among these, *Starmerella magnoliae* appears to play a key role during fermentation, as it has been consistently reported as the most abundant yeast present in bee bread [10,11,12,13,14]. The LAB community in bee bread comprises fructophilic species such as *Apilactobacillus kunkeei* and *Fructobacillus fructosus*, which preferentially metabolize fructose, followed by *Lactiplantibacillus plantarum*, *Levilactobacillus brevis*, and *Lactobacillus delbrueckii* subsp. *lactis* [15,16,17].

Several factors intrinsically influence the analytical composition of bee pollen, among which the most determining is botanical origin, since different plant species produce pollen grains with distinct nutritional profiles. Other influencing factors include climatic conditions, harvesting season, and storage practices [18]. Consequently, these factors influence bee bread composition; however, the fermentation process determines substantial biochemical changes that make it distinct from the original raw bee pollen. Carbohydrates, which account for 20–68% of bee pollen with fructose and glucose as the main sugars, are partially broken down during fermentation into simple sugars, which can be further metabolized into lactic acid, ethanol, mannitol, or short-chain fatty acids, enhancing both preservation and nutritional value [8,19,20,21]. The total phenolic content (TPC) in bee bread ranges from 0.14 to 69.53 mg GAE/g.

To date, most studies on bee pollen fermentation have mainly focused on endpoint comparisons between bee pollen and naturally fermented bee bread, providing valuable information on the compositional differences between the two matrices. However, the dynamic evolution of microbial communities during the transformation process remains less investigated. In particular, yeasts have received considerably less attention than lactic acid bacteria, despite their potential functional role in fermentation and their contribution to biochemical transformations occurring during bee bread formation [8,22,23,24].

The present study investigated the microbial dynamics, with particular emphasis on the changes and functional role of yeast populations, in five fresh bee pollen samples collected in the Marche Region (Italy) subjected to a fermentation process mimicking the natural transformation of bee pollen into bee bread. The evolution of lactic acid bacteria (LAB) was also monitored to provide a broader understanding of the fermentation process. The main characteristics of the resulting bee breads were subsequently evaluated for their chemical composition and nutritional features.

## 2. Materials and Methods

### 2.1. Samples and Spontaneous Fermentation

A total of five fresh bee pollen samples were analyzed. Four samples were purchased from local beekeepers in the Marche Region (Italy), originating from Fabriano (43°20′18.82″ N, 12°54′30.67″ E), Matelica (43°15′23.71″ N, 13°00′34.54″ E), Conero (43°33′07.2″ N, 13°36′16.92″ E), and Castel di Lama (42°52′10.39″ N, 13°42′47.88″ E). All samples were delivered and transported under frozen conditions (−20 °C) to the laboratory and processed immediately. A fresh bee pollen sample purchased from a chain of stores was included as a commercial sample, the unique officially available fresh bee pollen product in Italy.

Spontaneous fermentation was prepared according to Vamanu et al. [25]. Bee pollen, honey, and sterile distilled water were mixed at a ratio of 4:1:1 (*w*/*w*/*v*). Specifically, 120 g of unground bee pollen were combined with 30 g of honey and 30 mL of sterile distilled water. Each fermentation trial was performed in duplicate in sealed sterile glass jars to prevent evaporation and incubated at 27 °C for fourteen days as a compromise between mesophilic microbial growth conditions and the heterogeneous thermal environment of honeybee colonies [25,26].

### 2.2. Microbial Consortium Evolution During Bee Pollen Fermentation

To investigate the dynamics of the cultivable microbial community during the process, viable cell counts were performed by serial decimal dilution and plating on Wallerstein Laboratory (WL) nutrient agar (LIOFILCHEM^®^ S.r.l., Roseto degli Abruzzi, Teramo, Italy) for yeasts and molds and on de Man Rogosa Sharpe (MRS) agar (LIOFILCHEM^®^ S.r.l., Roseto degli Abruzzi, Teramo, Italy), a selective medium commonly used for the isolation and enumeration of LAB [27]. Presumptive LAB colonies were selected based on their colony morphology on MRS agar supplemented with 0.002% (*w*/*v*) cycloheximide to inhibit fungal growth, purified by subculturing, and subsequently subjected to identification analyses. WL nutrient agar plates were incubated at 25 °C for 2 days, whereas MRS agar plates were incubated at 30 °C for 3 days under microaerophilic conditions prior to colony-forming unit (CFU) enumeration. Viable counts were performed in duplicate for each sample with an interval of every 48 h during fermentation. To estimate the composition of the yeast and lactic acid bacteria (LAB) communities, the relative abundance (%) of each identified genus was calculated at each sampling point. Rather than relying on the raw number of isolated colonies, the relative abundance was determined based on the quantitative contribution of each identified taxon to the total viable count (CFU/g) on the respective selective media.

### 2.3. Yeasts and Lactic Acid Bacteria Identification

Preliminary identification was performed based on macro- and micro-morphological characteristics, which were monitored throughout the whole fermentation process. Selected yeast and LAB colonies, identified through morphological analysis, were purified on Yeast Peptone Dextrose (YPD) agar (1% yeast extract, 2% glucose, 2% peptone, and 2% agar; Oxoid, Basingstoke, UK) and MRS agar, respectively. For long-term storage, isolates were cryopreserved at −80 °C in YPD or MRS broth supplemented with 20% (*v*/*v*) glycerol.

The DNA of isolated yeasts was extracted according to the method described by Stringini et al. [28]. The ITS1- 5.8S rRNA-ITS2 region was amplified by PCR for yeast identification following the procedure reported by Agarbati et al. [11].

The DNA of the bacterial strains was extracted using the NucleoSpin Soil DNA, RNA, and protein purification kit (Macherey-Nagel, Düren, Germany). Amplification of the 16S rRNA gene was performed following the procedure reported by Taccari et al. [29]. The primer sets P1 (5′-CCTACGGGAGGCAGCAG-3′) and P2 (5′–ATTACCGCGGCTGCTGG-3′) were used, and the PCR products were separated by horizontal electrophoresis (Bio-Rad, Hercules, CA, USA) in 1.5% (*w*/*v*) agarose gel using 0.5× TBE buffer.

Amplicons were subjected to Sanger sequencing, and all obtained DNA sequences were compared with those existing in the GenBank database using the BLAST program (https://blast.ncbi.nlm.nih.gov/Blast.cgi, accessed in 1 October 2025 [30] and were deposited in the NCBI database under accession numbers PZ240740 to PZ240772.

### 2.4. Humidity and pH Monitoring

Moisture content was determined following the AOAC Official Method, as used by Anjos et al. [31], with modifications. Fresh bee pollen was weighed in a pre-weighed glass plate, dried at 75 °C for 24 h, and then reweighed. pH determination was carried out using a pH meter (LLG—pH METER 5; Lab Logistics Group GmbH, Meckenheim, Germany).

### 2.5. Protein and Reducing Sugars Determination

Reducing sugars were determined using the 3,5-dinitrosalicylic acid (DNS) method [32]. Briefly, 1 mL of sample or its dilutions was mixed with 2 mL of DNS reagent, heated in a boiling water bath at 100 °C for 5 min, and allowed to cool to room temperature; then the absorbance was measured at 540 nm. Sugar concentrations (g/100 g) were calculated using a glucose standard calibration curve.

Protein content was determined following the Lowry method as described by Waterborg et al. [33]. A 500 µL aliquot of the sample was mixed with 500 µL of Lowry reagent A and incubated at room temperature for 10 min. Subsequently, 250 µL of Lowry reagent B was added, followed by incubation in the dark for 30 min. Absorbance was measured at 750 nm, and the protein concentration (g/100 g) was quantified using a calibration curve constructed with bovine serum albumin (BSA) as the standard.

### 2.6. Total Polyphenol Content, Antioxidant Activity

Total polyphenol content, antioxidant activity, and antimicrobial activity were determined using the methanolic extract. Methanol extraction was carried out using the following method: two grams of sample were mixed with 20 mL of 80% (*v*/*v*) methanol and then placed under agitation at 180 rpm for 24 h at room temperature. After extraction, the mixture was filtered twice, first through filter paper and then through a 0.22 µm membrane filter. The resulting methanolic extract was stored at 4 °C for up to two weeks [22].

The total polyphenols content was determined using the Folin–Ciocalteu method [34].

Antioxidant activity was evaluated by the DPPH method [35]. Antioxidant activity was calculated using the following formula:

$$\mathrm{Antioxidant}\;\mathrm{activity}\;(\%)=\frac{\left(1-{\mathrm{Abs}}_{\mathrm{sample}}\right)}{{\mathrm{Abs}}_{\mathrm{blank}}}\times 100$$

### 2.7. Antimicrobial Activity

Antimicrobial activity was evaluated following a modified method described by Kaškonienė et al. [22]. The pathogens used were *Staphylococcus aureus*, *Escherichia coli*, *Listeria monocytogenes*, *Salmonella enterica,* and *Candida albicans*. Pathogen precultures were grown in modified plate count (PC) broth (peptone 2.5 g/L, yeast extract 1.25 g/L, glucose 0.5 g/L) for 24 h at 37 °C. A 10^5^ CFU/mL of each pathogen was incorporated into 20 mL of Nutrient Agar (meat extract 3 g/L, peptone 5 g/L, agar 15 g/L). Controls were prepared as follows: a positive control with 5% chloramphenicol, a negative control with 0.9% NaCl, and a solvent control using 80% methanol. After approximately 30 min to allow agar solidification, wells were carefully removed, and 80 µL of extract was added to each well. Plates were incubated at 37 °C for 24 h. Antimicrobial activity was evaluated by measuring the diameter (mm) of the inhibition halo (reduction in growth) or clarification halo (kill action) around the well.

### 2.8. Evaluation of Bee Pollen

Pollen grain integrity was assessed using a modified method described by Campos et al. [36]. Glycerin jelly stain was prepared by dissolving 10 g of gelatin in 34.2 mL of deionized water under agitation for 2 h, followed by the addition of 66.3 g of glycerol, 0.5 g of carbonic acid, and 0.1 g of safranin, and stored at 4 °C. For slide preparation, 50 µL of the 1:10 diluted pollen was placed on a microscope slide and dried on a heated plate, then 30 µL of glycerin jelly was added and covered with a coverslip; slides were sealed with transparent nail polish after one week. Slides were observed under a 10× and 40× objective, photographs were taken with Leica ICC50 HD (Leica Microsystems, Wetzlar, Germany), and pollen grains were analyzed using ImageJ software (version 1.53t; National Institutes of Health, Bethesda, MD, USA) to assess their integrity and morphology, as well as for quantitative counting.

### 2.9. Statistical Analysis

All experimental fermentations were carried out in independent biological duplicates. For each biological replicate, all subsequent microbiological and physicochemical measurements were performed in technical triplicate. For statistical analysis, data were expressed as the mean value ± standard deviation of the technical replicates across the biological trials. All experimental data were subjected to statistical analysis using RStudio software (version 2022.12.0; Posit Software, PBC, Boston, MA, USA) [37]. A two-way ANOVA was performed to evaluate significant differences among means, followed by Duncan’s multiple range test for post hoc comparisons, with the significance level set at *p*-value < 0.05. Additionally, principal component analysis (PCA) was applied to evaluate the overall biochemical shifts and discriminate the bee bread samples based on the fermentation time. Prior to multivariate analysis, the dataset was centered and scaled via Z-score normalization to ensure that all the physicochemical variables contributed equally to the model, regardless of their original measurement scales and units.

## 3. Results

### 3.1. Spontaneous Fermentation of Bee Pollen

**Figure 1:**
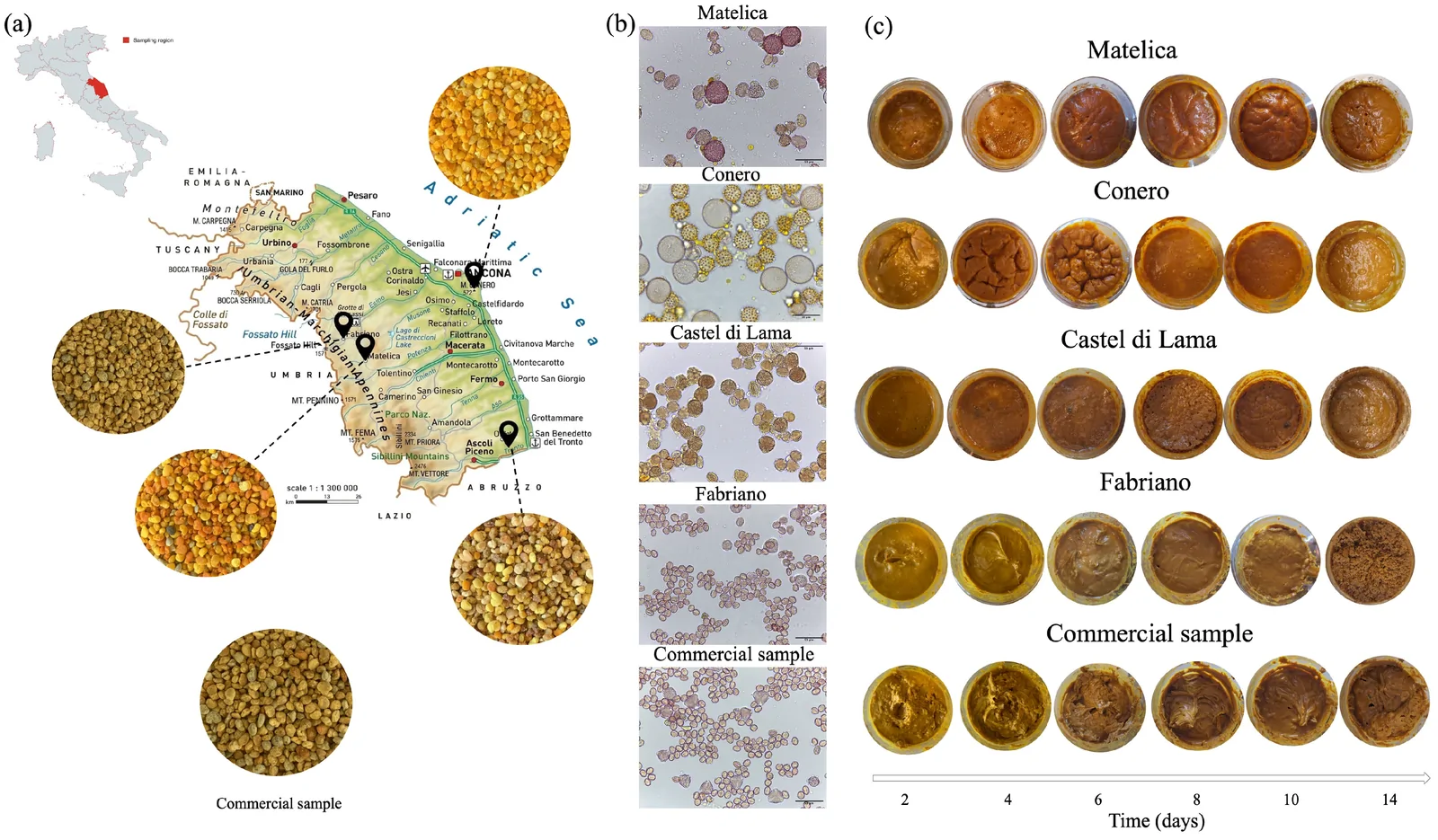
Geographical origin of the bee pollen samples, microscopic morphology of pollen grains, and macroscopic evolution of spontaneous bee bread fermentation from different pollen samples: (**a**) geographical origin of bee pollen samples and macroscopic characteristics; (**b**) microscopic morphology of pollen (40×), stained with modified glycerin jelly; (**c**) macroscopic evolution of bee pollen during fermentation.

Figure 1 illustrates the geographical origins of bee pollen samples, the microscopic analysis of pollen grains, and macroscopic changes occurring during spontaneous bee bread fermentation.

Macroscopic observation revealed clear visual differences among the pollen samples, particularly in the color and appearance of the individual pollen pellets. These differences were further confirmed by microscopic analysis, which highlighted distinct pollen grain morphologies among the samples. Conversely, Fabriano and commercial bee pollen samples appeared highly similar both macroscopically and microscopically.

Among the analyzed samples during fermentation, Matelica and Conero bee pollens showed the most pronounced swelling. Matelica bee pollen reached its maximum swelling on the sixth day, after which it gradually deflated, acquiring a creamy appearance. In contrast, Conero bee pollen exhibited a more gradual swelling pattern, reaching its maximum expansion on the 10th day, followed by deflation observed on the 14th day. Castel di Lama bee pollen did not show a clear swelling phase; instead, it developed a spongy bee bread structure, with the maximum expression of this characteristic observed on the eighth day. A similar spongy structure was observed in Fabriano bee pollen, although this occurred later, reaching its peak on the last day of fermentation.

No evident swelling or major structural transformation was observed in the commercial pollen during fermentation; instead, the primary macroscopic change was a progressive homogenization of the matrix. Overall, spontaneous fermentation displayed distinct macroscopic evolutionary patterns depending on pollen origin, suggesting a potential relationship between fermentation behavior and the intrinsic microbial communities associated with each sample. Nevertheless, some similarities could be identified, particularly between Matelica and Conero bee pollens, as well as between Fabriano and Castel di Lama, which displayed comparable fermentation patterns.

### 3.2. Microbial Consortium Evolution During Bee Bread Fermentation

**Figure 2:**
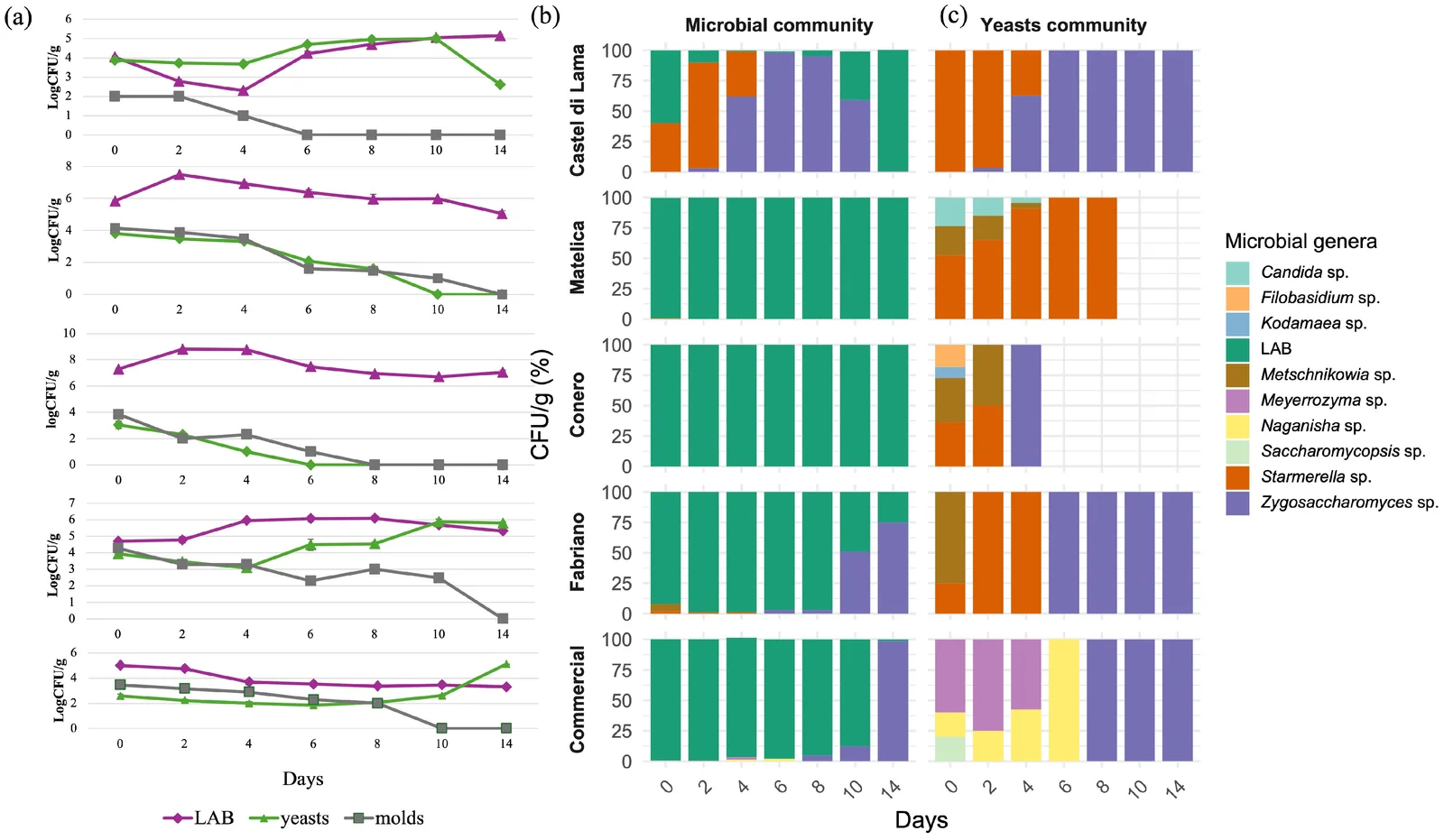
Microbial population dynamics during spontaneous bee bread fermentation in five varieties of fresh bee pollen. (**a**) Microbial kinetics during spontaneous fermentation expressed as log CFU/g, including lactic acid bacteria (LAB), yeasts, and molds; (**b**) estimated relative abundance (%) of cultivable microbial communities based on viable counts; (**c**) evolution of cultivable yeast communities during spontaneous fermentation. Data means ± standard deviations are represented as error bars.

The evaluation of microbial kinetics and the relative abundance of microorganisms across the five bee pollen samples during spontaneous fermentation into bee bread is reported in Figure 2. The analysis revealed distinct microbiological trends among the individual samples, which were associated with different pollen grain morphologies reflecting their distinct geographical origins, as shown in Figure 1b. In the Conero and Matelica samples, the abundant initial presence of lactic acid bacteria (Log 7.3 and 5.8 CFU/g, respectively) determined an inhibition of yeast development. The yeasts, which start from lower concentrations compared to LAB (Figure 2), showed a progressive reduction, leading to their complete disappearance within a few days. In both cases, LAB reached their maximum concentration on the second day of fermentation, when the yeasts were still present in the substrate. Conversely, in the Castel di Lama sample, in which the initial concentrations of LAB (Log 4.0 CFU/g) and yeasts (Log 3.9 CFU/g) were comparable, an opposite trend was observed. Indeed, a marked predominance of yeasts over bacteria until the 10th day, with peaks of their respective populations (Log 5.0 CFU/g). A further distinct situation was observed in the bee bread generated from Fabriano and commercial bee pollen. Although LAB dominated the fermentation, both microbial populations increased simultaneously and uniformly, especially during the later stages of fermentation. By the end of fermentation, yeasts reached higher concentrations than lactic acid bacteria in both samples of bee bread, with Log 5.3 CFU/g and Log 5.1 CFU/g in the Fabriano and commercial samples, respectively. Overall, yeasts predominated within the microbial community at the 14th day of fermentation, accounting for 75% and 98% of the total cultivable population in the Fabriano and commercial samples, respectively, as reported in Figure 2b. All fermentation processes showed the complete elimination of potential spoilage molds in the final products.

### 3.3. Microbial Population Analysis

A total of 36 yeasts and 13 LAB were identified (Table 1). The most prevalent lactic acid bacterium was *A. kunkeei*, confirming its widespread presence in the honeybee ecosystem. Notably, *A. kunkeei* was consistently detected in all analyzed samples throughout the whole fermentation process. In contrast, *F. fructosus* was detected at low abundance and only at the beginning of the fermentation in the bee pollen sample from Castel di Lama, suggesting its marginal role.

Among the yeasts detected during the fermentation process, the genera *Zygosaccharomyces* and *Starmerella* were found in four out of five samples, suggesting their potential role as active contributors to the fermentation process. Indeed, both genera shared a preference for fructose over glucose as a carbon source, making them metabolically like the fructophilic lactic acid bacteria group, which likely confers a selective advantage in bee pollen, a substrate characterized by a high fructose/glucose ratio.

The genus *Zygosaccharomyces* represented the most heterogeneous and isolated group, comprising three distinct species. The most abundant species was *Z. rouxii*, followed by *Z. siamensis* and *Z. mellis,* which was detected twice at the late stage of fermentation in bee pollen from Castel di Lama and Fabriano. Yeasts belonging to the genus *Zygosaccharomyces* appeared in the middle of the fermentation and persisted until the end, driving the late-stage fermentation. Conversely, several species of the genus *Starmerella* were detected early, with *S. magnoliae* the most abundant, followed by *S. bombicola* and *Starmerella* sp. *Starmerella* genus was present from the beginning of fermentation and up to the midpoint, showing a decreasing trend toward the later stages. Indigenous flower-associated yeasts were also identified, including *Saccharomycopsis capsularis*, *Filobasidium magnum*, *Metschnikowia pulcherrima*, *Kodamaea ohmeri*, *Candida cetoniae*, *Meyerozyma guilliermondii*, *Naganishia* *albida* and *N. bhutanensis*.

The low abundance of these yeasts suggests that during the early stages of fermentation, the cultivable microbiota remains heterogeneous and distributed across different ecological niches, namely the floral environment and the beehive-associated environment.

### 3.4. Analytical Characters of Bee Bread Samples

**Figure 3:**
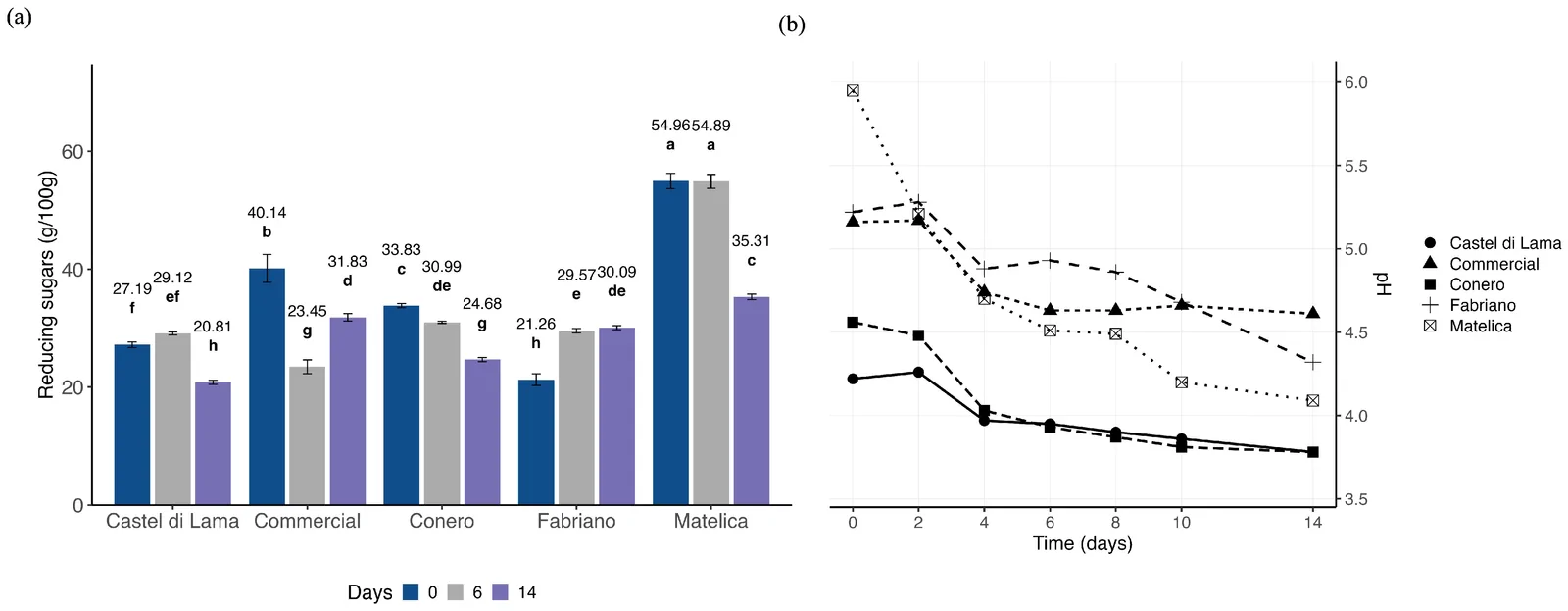
Variation in reducing sugars and pH during fermentation: (**a**) reducing sugar content (g/100 g) at days 0, 6, and 14 of fermentation, and (**b**) pH monitored throughout the fermentation process. Values are shown with error bars. Different superscript letters indicate significant differences among samples and fermentation times according to Duncan’s test (*p* < 0.05).

The initial moisture content as well as the reducing sugar concentration and pH value varied among the samples. The moisture content ranged from 13.5% in the Fabriano sample to 21.8% in the Conero sample, with intermediate values recorded for the Matelica (13.8%), Commercial bee pollen (16.7%), and Castel di Lama (18.4%) samples. The trends of reducing sugars and pH over time are shown in Figure 3. In general, the samples showed a significant reduction in reducing sugars, followed by a decrease in pH during fermentation, confirming the crucial role of microbiota in the production of metabolites from sugars, such as lactic acid. Overall, a decrease in reducing sugars was observed in the final product, except for the bee bread derived from Fabriano, in which an increase in reducing sugars of 7.9 g/100 g was recorded between the beginning and the midpoint of fermentation. The highest consumption of reducing sugars was observed in the Matelica sample (19.1 g/100 g), which decreased after the 6th day of fermentation, followed by the Conero sample, which showed a gradual reduction to 9.14 g/100 g. In contrast, in the commercial sample fermentation, an initial decrease in reducing sugars was observed (15.9 g/100 g), followed by a subsequent increase from the 6th day of fermentation until the end, reaching a final concentration of 31.8 g/100 g.

A different trend was observed in the Castel di Lama sample, where an initial increase in reducing sugars was detected until the midpoint of fermentation, followed by a decrease, with a final value of 20.8 g/100 g. All samples showed a consistent pH reduction of around one point. The sample that showed the greatest decrease in pH was Matelica, followed by Fabriano and Conero, whereas the commercial sample and the Castel di Lama sample exhibited the smallest variation in pH but also the lowest starting point.

### 3.5. Quantification of Proteins and Bee Pollen Disruption

**Figure 4:**
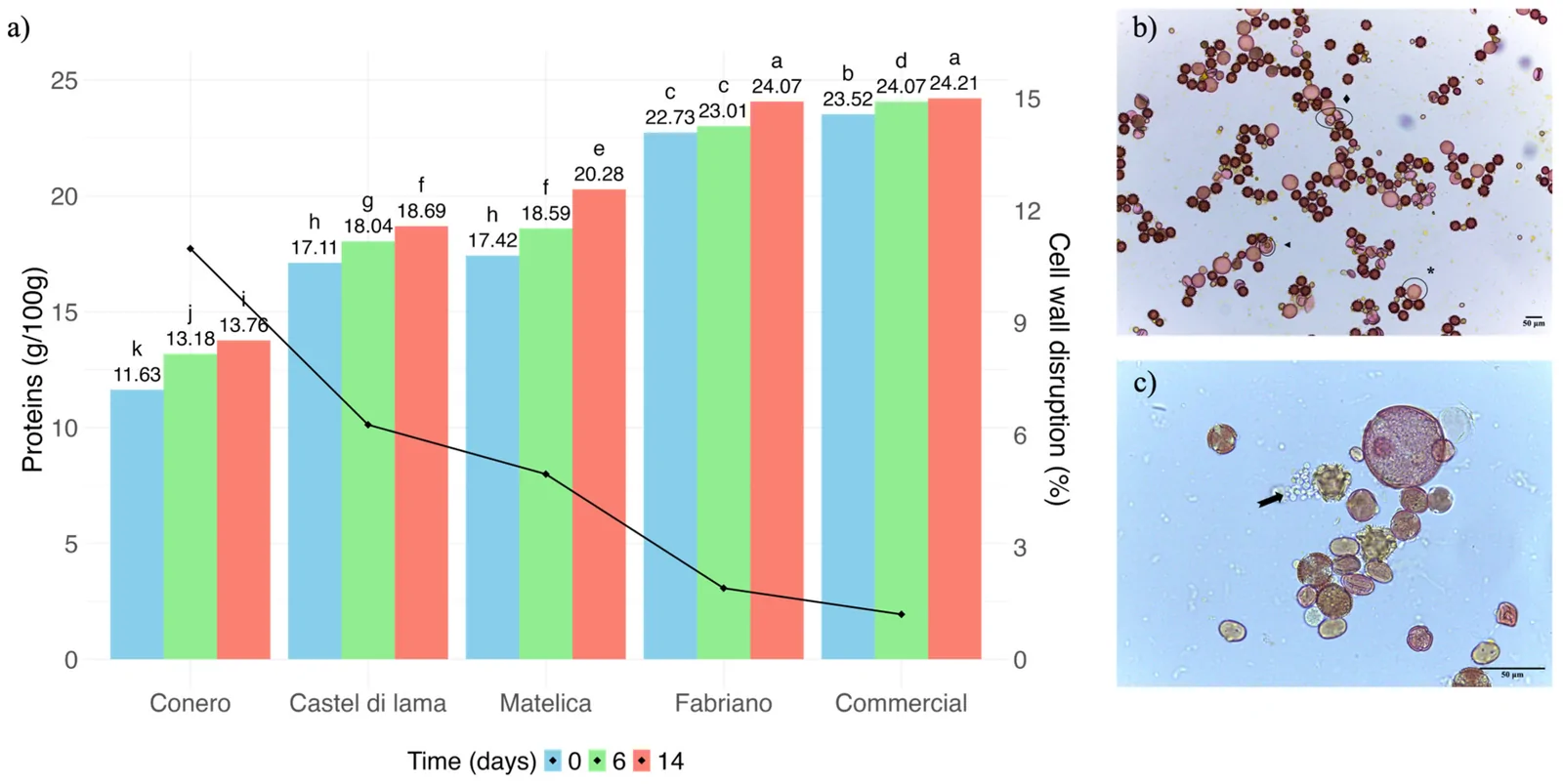
Integrated analysis of protein content, pollen wall disruption, and microbial activity during fermentation. (**a**) Relationship between protein content and cell wall disruption (%); (**b**) 10× micrograph of the Conero sample after fermentation with intact bee pollen (*), bee pollen depleted of its cytoplasmatic content (♦), and completely degraded bee pollen (◄); (**c**) microbial attack (➞) by a cluster of yeasts on a bee pollen of the *Asteraceae* family from Castel di Lama. Superscript letters indicate significant differences among samples and fermentation times according to Duncan’s test (*p* < 0.05).

The protein content over time in the different samples and the percentage of bee pollen disruption are reported in Figure 4a. The protein content of bee pollen varies among the samples, reflecting the difficulty of standardizing the product due to the diverse local flora present in each geographical sampling. The commercial sample had the highest protein content with an amount of 23.52 g/100 g, while the sample from Conero showed the lowest protein profile (11.63 g/100 g).

An overall increase in total protein content can be observed during the process.Indeed, a significant increase in protein concentration at the end of the fermentation was shown in all samples. The greatest increase in protein content was observed in the Conero sample, which exhibited an increase of 18%. This sample also showed the best microbial performance in cell wall pollen grain degradation, followed by Matelica (16%) and Castel di Lama (9%). Among these latter two samples, an interesting difference emerged. Interestingly, although the Matelica and Castel d i Lama samples exhibited comparable initial protein contents (17.42 and 17.11 g/100 g, respectively), the final protein concentration was higher in the Matelica sample (20.28 g/100 g) than in the Castel di Lama sample (18.69 g/100 g). This finding suggests a more efficient release of proteins during fermentation in the Matelica sample, likely reflecting differences in microbial activity or pollen grain susceptibility to degradation. The progressive stages of pollen grain degradation, characterized by the transition from intact grains to those progressively depleted of their cytoplasmic content until only the empty outer shell remained, are clearly visible in the microscopic observations reported in Figure 4b. Conversely, Fabriano and the commercial samples showed lower protein release from bee pollen, corresponding to a lower percentage of cell wall degradation, 2.38% and 1.16%, respectively. Notably, there was a correlation between protein content and the microbial activity toward pollen grains. As illustrated in Figure 4c, this microbial interaction leads to a structural collapse by breaking the pollen grain wall, which promotes the leakage of intracellular nutrients and the subsequent increase in free proteins in the medium.

### 3.6. Total Polyphenol Content and Antioxidant Activity

The variation in total polyphenols content and antioxidant activity before and after fermentation is shown in Table 2. The total polyphenols content was significantly increased in Castel di Lama, Matelica, and in the commercial sample after fermentation, whereas a slight decrease was detected in Fabriano and Conero samples. The antioxidant activity of the product, being a combination of bee pollen and honey, was already high, ranging from 89.35% to 90.96%. This high antioxidant activity was maintained after fermentation in all samples, without significant differences from the beginning to the end of the process, while the Matelica sample showed a significant increase in antioxidant activity after fermentation.

**Figure 5:**
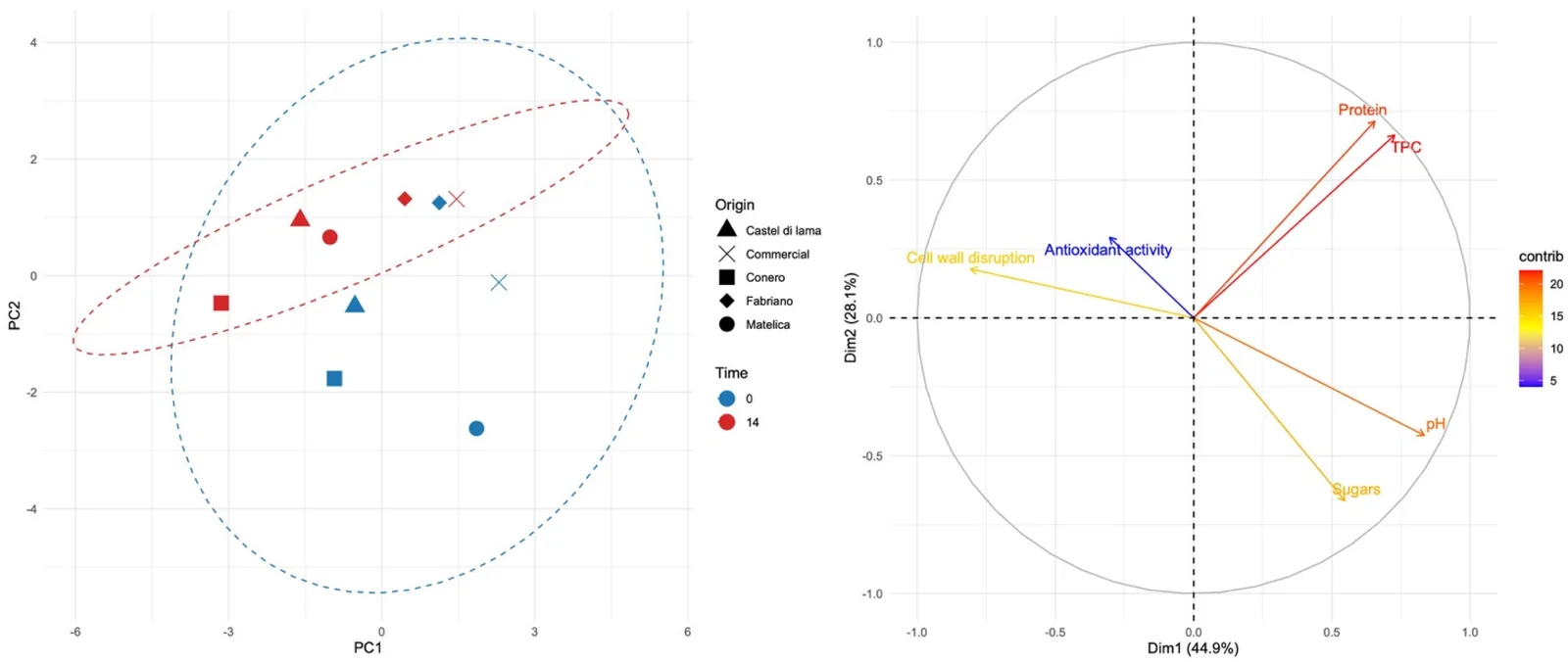
Principal component analysis (PCA) of bee bread biochemical profiles. The variance explained by PCA is PC1 = 44.9% on the x-axis and PC2 = 28.1% on the y-axis. Blue and red symbols represent samples at 0 and 14 days of fermentation, respectively, while shapes indicate the samples.

The principal component analysis (PCA) clearly highlighted the biochemical evolution of the samples during the fermentation process, explaining 73% of the total variance. Along the PC1 axis, a distinct directional shift was observed for all geographical origins. This transition was driven by the decrease in pH and sugar content, coupled with an increase in cell wall disruption. Interestingly, the Fabriano sample represented a notable exception, as its unfermented (Time 0) and fermented (Time 14) forms clustered closely together, suggesting that its biochemical profile did not undergo substantial changes during the process. Nevertheless, although differences in geographical origin are known to influence the basal nutritional profile of bee pollen, the fermentation process exerted a converging effect. Fermented samples clustered more closely together compared to their unfermented counterparts. This biochemical convergence demonstrates that, despite the initial heterogeneity of the raw bee pollen, the standardized action of microbial fermentation successfully aligns the final products towards a uniform, nutritionally enhanced, and functional bee bread profile (Figure 5).

### 3.7. Antimicrobial Activity

The antimicrobial activity of individual bee pollen samples tested before and after fermentation, against four pathogenic bacterial strains (*E. coli*, *L. monocytogenes*, *S. aureus*, and *S. enterica*) and the fungal pathogen *C. albicans* is reported in Table 3.

The results showed that the antimicrobial activity was enhanced after fermentation. Notably, Matelica and Fabriano bee pollen showed a marked increase in activity against *S. aureus*; in both cases, the fermentation of the samples determined complete inhibition. A similar effect, although to a lesser extent, was observed for Conero and the commercial sample, where fermentation did not lead to complete inhibition but still caused a significant reduction in bacterial growth compared to the unfermented products.

The bee pollen samples were also highly effective against *L. monocytogenes*. The unfermented substrate already induced partial inhibition, which turned into complete growth inhibition after fermentation. Matelica bee pollen shifted from a slight reduction in bacterial growth with the unfermented extract to complete inhibition following fermentation, determining an inhibition halo of 21 ± 0.71 mm. Additionally, all bee pollen samples exhibited increased growth reduction but not an inhibition halo against the enteric pathogen *E. coli* after fermentation. In contrast, *S. enterica* was more resistant to both unfermented and fermented bee pollen, showing only limited antimicrobial activity. A slight enhancement of growth reduction (already observed with unfermented pollen) was shown in the Castel di Lama and Commercial samples. Fabriano and the commercial samples before fermentation were the only substrates able to induce a slight reduction in the growth of the yeast pathogen *C. albicans*.

## 4. Discussion

Bee pollen contains more than 250 bioactive molecules, which places it within the category of “superfoods” [38]. However, it is also well known that, due to its physicochemical composition, humans cannot fully digest bee pollen and therefore completely benefit from its nutritional properties [24,39,40,41,42,43,44].

An effective way to improve the digestibility of bee pollen has been developed by honeybees. Inside the hive, honeybees initiate a fermentation process that transforms raw bee pollen into bee bread. Through the breakdown of pollen grains, resulting from the combined action of fermentative microorganisms and honeybees’ enzymes, pollen is converted into a product with higher nutrient bioavailability. Moreover, bee bread acts as a symbiotic food for honeybees’ larvae, providing them with the appropriate “microbial imprinting” and essential nutrients needed for proper development [45].

This study investigated the dynamics over time of the microbial consortium during the fermentation of bee pollen samples, with a specific focus on yeast population succession.

The results suggested that fermentation is driven by a relatively constant microbial community, independent of the geographical origin of the pollen, characterized by the recurrent presence of yeasts belonging to the genera *Zygosaccharomyces* and *Starmerella*, detected in four out of five fermentations and consistently associated with the lactic acid bacterium *A. kunkeei.* Additionally, yeasts belonging to the *Metschnikowia* genus were isolated at the beginning phase in three out of five samples, suggesting a possible role at this stage. Among the microbial members of the stable consortium, *A. kunkeei* is recognized as a key component of the honeybee core gut microbiota, supporting the hypothesis that it may contribute probiotic functions within bee bread [17,24,46,47]. Regarding yeasts, both *Zygosaccharomyces* and *Starmerella* genera share fructophilic traits and grow in high-sugar ecological niches such as honeybee habitats [48,49,50,51,52,53]. A recent study confirmed the presence of several *Zygosaccharomyces* species in honeybee-associated environments and identified *Z. rouxii*, primarily isolated from larval samples, as the main ergosterol-producing species, whose ergosterol can be utilized by *Apis mellifera* larvae as a precursor of molting hormones [54]. Consistent with these observations, our results showed that *Z. rouxii* was the dominant species found within this genus. Moreover, the marked increase in *Zygosaccharomyces* during the later stages of fermentation may enhance ergosterol availability in mature bee bread, coinciding with the period in which larvae consume this food source.

Since the first microbiological studies on the bee environment, yeasts belonging to the genus *Starmerella* have consistently been detected in bee pollen, bee bread, and the bee gut [10,55,56] but their role within this system has not yet been fully elucidated [12,57,58,59].

As suggested by Gilliam [10] and supported by Agarbati et al. [12], *S. magnoliae*, one of the most frequently isolated *Starmerella* species in the honeybee ecosystem, may represent part of the stable mycobiota of honeybees. Its consistent presence in bee bread and bee pollen, together with its limited detection in flowers, suggests that this yeast may be directly inoculated on bee pollen before fermentation.

A hypothetical role of *Starmerella* species during pollen fermentation may involve their interaction with pollenkitt through the production of sophorolipids, biosurfactant molecules known to be synthesized by members of this genus [14]. Our results showed that an increasing degree of pollen grain rupture related to the presence of *Starmerella* species may support this hypothesis.

*Metschnikowia* species are more commonly associated with flowers and floral pollen than with mature bee bread [60]. Among them, *M. pulcherrima* is a well-known nectar yeast involved in plant–pollinator interactions [61] and has also demonstrated antimicrobial activity against the honeybee pathogen *Ascosphaera apis* [62]. This property may help explain how, despite its environmental origin, *M. pulcherrima* may have progressively established a symbiotic relationship with honeybees. Consistent with these results, *Metschnikowia* was detected in three out of five samples, exclusively during the early stage of fermentation. Other yeasts commonly associated with floral microbiota were also identified, including *F. magnum*, *K. ohmeri*, *C. cetoniae*, *M. guilliermondii*, *N. albida,* and *N. bhutanensis*. Each of these taxa was recovered only once, suggesting a transient environmental contribution rather than stable persistence within the fermentative process [63,64,65,66]. The observed microbial succession is driven by a fermentation-induced shift in physicochemical conditions, where initial *Starmerella* sp. activity facilitates lactic acid bacteria establishment [67,68]. Subsequently, LAB activity, particularly *A. kunkeei*, drives a pH reduction below 4.0, which suppresses sensitive floral yeasts, while pollen hydrolysis simultaneously increases sugar availability and osmotic pressure, thereby selecting for highly tolerant taxa such as *Z. rouxii* [69]. Finally, molds were absent in all samples at the end of fermentation, supporting the hypothesis proposed by [70] that the cooperation of yeasts and lactic acid bacteria exerts an inhibitory effect against mold development. These findings further highlight the stabilizing role of the fermentation process.

An increase in the measured protein content was observed at the end of fermentation in all samples, likely reflecting improved protein extractability and bioavailability due to microbial activity [1,19,71]. This finding supports the essential role of fermentation in the enhancement of protein accessibility, thereby contributing to the nutritional requirements for proper colony development [72]. However, high protein concentrations seem to limit microbial enzymatic degradation of pollen walls. Interestingly, honeybees appear to account for this constraint during pollen collection, unlike bumblebees, which actively prefer pollen with higher protein content [73]. This difference may be associated with the fact that bumblebees do not produce bee bread but consume raw pollen directly [74]. Furthermore, samples from Castel di Lama and Conero showed higher microbial diversity, potentially providing a wider array of enzymatic pathways for pollen degradation, consistent with similarities between yeast enzymatic systems [10].

Changes in reducing sugar content during fermentation appeared to be sample-dependent. The greatest decrease in reducing sugars was observed in Matelica and Conero samples, where lactic acid bacteria dominated the entire fermentation process [1,75]. Nevertheless, the samples from Fabriano and Castel di Lama, as well as the commercial sample, showed an increase in reducing sugars, likely due to enzymatic hydrolysis of complex carbohydrates such as sucrose, maltose, and structural polysaccharides in the pollen wall [76,77]. In these samples, the progressive increase in *Z. rouxii* before the rise in reducing sugars is consistent with the known ability of *Zygosaccharomyces* species to utilize maltose and sucrose as carbon sources through the production of extracellular hydrolases [78,79].

Total polyphenol content in the analyzed bee pollen samples was generally lower than literature averages [22,23,80,81,82]. However, the obtained values are consistent with those reported by several authors [80,83,84] which described a concentration from 0.142 to 2.5 mg GAE/g.

This variability may be associated with several factors, including botanical origin, high moisture content in fresh pollen, and storage conditions, including freezing at −20 °C [85,86,87,88]. Consistently, bee bread produced from the Castel di Lama and Conero bee pollen samples, which exhibited the highest moisture content, showed the lowest polyphenol concentrations. However, in most samples, fermentation increased the total polyphenols content [22,89,90,91]. An enhancement of antioxidant capacity was also observed in bee bread produced from Matelica pollen, in line with the findings of Kaškonienė et al. [22]. In general, fermentation enhances the functional properties of the final product, particularly its antimicrobial activity. Indeed, bee bread exhibited stronger inhibition of pathogens compared to unfermented bee pollen [20,83,92]. Our results are consistent with those reported in the literature, as the tested Gram-positive bacteria, *S. aureus* and *L. monocytogenes*, showed higher sensitivity compared with Gram-negative pathogens such as *E. coli* and *S. enterica*, which are generally more resistant, likely due to the difference in the composition of the bacterial wall. Bee bread and bee pollen are rich in phenolic acids, flavonoids, and organic acids, which may contribute to similar bactericidal effects. In contrast, *Candida albicans* remained largely unaffected throughout fermentation [83,93].

## 5. Conclusions

In conclusion, the results of this study provide new information on the dynamics of yeast populations during bee bread formation, highlighting the occurrence and contribution of yeasts belonging to the genera *Zygosaccharomyces* and *Starmerella*. These yeasts, together with *A. kunkeei*, formed a stable cultivable microbial consortium associated with the biotransformations throughout fermentation, potentially contributing to improving product stability and the enhancement of its functional properties. However, further investigations are needed to set up a fermentation process using bee pollen and honey to produce a bee bread-like product with a stable, high-value functional food.

## Figures and Tables

**Table 1:** Microbial community evolution during the spontaneous fermentation of bee pollen into bee bread.

		Fermentation Progress (Days)		
Geographical Origin	Yeast Species	Start (0–5)	Middle (6–10)	End (11–14)
Castel di Lama	*Starmerella magnoliae*	2		
Castel di Lama	*Zygosaccharomyces mellis*		1	
Castel di Lama	*Zygosaccharomyces rouxii*	3	2	1
Castel di Lama	*Zygosaccharomyces siamensis*	1	1	1
Castel di Lama	*Apilactobacillus kunkeei*			1
Castel di Lama	*Fructobacillus fructosus*	1		
Conero	*Filobasidium magnum*	1		
Conero	*Kodamea ohomeri*	1		
Conero	*Metschnikowia pulcherrima*	1		
Conero	*Starmerella* sp.	2		
Conero	*Zygosaccharomyces siamensis*	1		
Conero	*Apilactobacillus kunkeei*	1		1
Fabriano	*Metschnikowia pulcherrima*	1		
Fabriano	*Starmerella magnoliae*		1	
Fabriano	*Zygosaccharomyces rouxii*		3	2
Fabriano	*Zygosaccharomyces mellis*			1
Fabriano	*Apilactobacillus kunkeei*	2	1	1
Matelica	*Candida cetoniae*	1		
Matelica	*Starmerella* sp.		1	
Matelica	*Metschnikowia* sp.	1		
Matelica	*Starmerella bombicola*	1		
Matelica	*Metschnikowia pulcherrima*	1		
Matelica	*Apilactobacillus kunkeei*	2	1	1
Commercial sample	*Meyerozyma guillermondii*	1		
Commercial sample	*Naganishia albida*	1		
Commercial sample	*Naganishia bhutanensis*		1	
Commercial sample	*Saccharomycopsis capsularis*	1		
Commercial sample	*Zygosaccharomyces rouxii*			1
Commercial sample	*Apilactobacillus kunkeei*			1

**Table 2:** Antioxidant activity (%) and polyphenol content (mg GAE/g) before and after spontaneous fermentation process.

	Antioxidant Activity (%)		Polyphenol Content (mg GAE/g)	
	Before	After	Before	After
Conero	90.72 ± 0.10 ^a^	90.26 ± 0.69 ^a^	0.54 ± 0,01 ^a^	0.38 ± 0.01 ^b^
Castel di Lama	90.07 ± 0.64 ^a^	90.42 ± 0.25 ^a^	0.85 ± 0,02 ^b^	1.33 ± 0.01 ^a^
Matelica	90.26 ± 0.05 ^b^	92.00 ± 0.20 ^a^	1.03 ± 0,03 ^b^	1.25 ± 0.03 ^a^
Fabriano	90.96 ± 0.25 ^a^	90.98 ± 0.10 ^a^	2.11 ± 0,02 ^a^	1.88 ± 0.01 ^b^
Commercial	89.35 ± 0.15 ^a^	90.26 ± 0.40 ^a^	2.17 ± 0,01 ^b^	2.54 ± 0.02 ^a^

Values are the mean ± standard deviation. Superscript letters (a, b) indicate significant differences within each sample according to Duncan’s test (*p* < 0.05).

**Table 3:** Antimicrobial activity of bee pollen expressed as the diameter of inhibition (I) or reduction (R) halos (mm) before and after fermentation against *E. coli*, *S. enterica*, *S. aureus*, *L. monocytogenes*, and *C. albicans*. Ctr + 5% chloramphenicol in ethanol; ctr -:0.9% NaCl.

Halo (mm)										
	Matelica		Castel di Lama		Conero		Fabriano		Commercial	
	Before	After	Before	After	Before	After	Before	After	Before	After
*E. coli*	14.0 ± 1.41 ^c^ R	16.5 ± 0.71 ^b^ R	15.5 ± 0.71 ^bc^ R	21.0 ± 0.00 ^a^ R	19.5 ± 0.71 ^a^ R	21.0 ± 0.00 ^a^ R	16.5 ± 0.71 ^b^ R	17.0 ± 0.00 ^b^ R	14.0 ± 1.41 ^c^ R	20.0 ± 1.41 ^a^ R
*S. enteritis*	19.5 ± 0.71 ^bcd^ R	15.0 ± 0.00 ^ef^ R	14.0 ± 1.41 ^ef^ R	22.0 ± 1.41 ^a^ R	18.0 ± 0.00 ^d^ R	13.0 ± 0.00 ^f^ R	20.5 ± 0.71 ^abc^ R	18.5 ± 0.71 ^cd^ R	16.0 ± 1.41 ^e^ R	21.0 ± 0.00 ^ab^ R
*S. aureus*	0.0 ± 0.00 ^e^	14.0 ± 1.41 ^a^ I	9.0 ± 1.41 ^c^ I	12.0 ± 0.00 ^b^ I	0.0 ± 0.00 ^e^	4.0 ± 1.41 ^d^ R	0.0 ± 0.00 ^e^	10.0 ± 1.41 ^c^ I	0.0 ± 0.00 ^e^	1.0 ± 0.00 ^e^ R
*L. monocytogenes*	8.0 ± 1.41 ^e^ R	21.5 ± 0.71 ^a^ I	10.0 ± 0.00 ^de^ R	13.0 ± 0.00 ^b^ I	13.0 ± 1.41 ^b^ R	13.0 ± 1.41 ^b^ I	10.5 ± 0.71 ^cd^ R	12.5 ± 0.71 ^bc^ I	10.5 ± 0.71 ^cd^ R	13.5 ± 0.71 ^b^ I
*C. albicans*	0.0 ± 0.00 ^b^	0.0 ± 0.00 ^b^	0.0 ± 0.00 ^b^	0.0 ± 0.00 ^b^	0.0 ± 0.00 ^b^	0.0 ± 0.00 ^b^	10.0 ± 0.00 ^a^ R	0.0 ± 0.00 ^b^	10.0 ± 0.00 ^a^ R	0.0 ± 0.00 ^b^

Values are expressed as mean ± standard deviation. Superscript letters (a–f) indicate significant differences among means according to Duncan’s test (*p* < 0.05). Positive and negative controls were used to validate the assay and were not included in the statistical analysis.

## Data Availability

The original contributions presented in this study are included in the article. Further inquiries can be directed to the corresponding authors.
